# Predicting Hospitalization in Older Adults Using Machine Learning

**DOI:** 10.3390/geriatrics10010006

**Published:** 2025-01-04

**Authors:** Raymundo Buenrostro-Mariscal, Osval A. Montesinos-López, Cesar Gonzalez-Gonzalez

**Affiliations:** School of Telematics, University of Colima, Colima 28040, Mexico; oamontes@ucol.mx (O.A.M.-L.); cgonzalez31@ucol.mx (C.G.-G.)

**Keywords:** hospitalization, older adults, health prediction, machine learning, random forest

## Abstract

**Background/Objectives**: Hospitalization among older adults is a growing challenge in Mexico due to the high prevalence of chronic diseases and limited public healthcare resources. This study aims to develop a predictive model for hospitalization using longitudinal data from the Mexican Health and Aging Study (MHAS) using the random forest (RF) algorithm. **Methods**: An RF-based machine learning model was designed and evaluated under different data partition strategies (ST) with and without variable interaction. Variable importance was assessed based on the mean decrease in impurity and permutation importance, enhancing our understanding of predictors of hospitalization. The model’s robustness was ensured through modified nested cross-validation, with evaluation metrics including sensitivity, specificity, and the kappa coefficient. **Results**: The model with ST2, incorporating interaction and a 20% test proportion, achieved the best balance between sensitivity (0.7215, standard error ± 0.0038), and specificity (0.4935, standard error ± 0.0039). Variable importance analysis revealed that functional limitations (e.g., abvd3, 31.1% importance), age (12.75%), and history of cerebrovascular accidents (12.4%) were the strongest predictors. Socioeconomic factors, including education level (12.08%), also emerged as critical predictors, highlighting the model’s ability to capture complex interactions between health and socioeconomic variables. **Conclusions**: The integration of variable importance analysis enhances the interpretability of the RF model, providing novel insights into the predictors of hospitalization in older adults. These findings underscore the potential for clinical applications, including anticipating hospital demand and optimizing resource allocation. Future research will focus on integrating subgroup analyses for comorbidities and advanced techniques for handling missing data to further improve predictive accuracy.

## 1. Introduction

Hospitalization among older adults is a critical public health issue in Mexico. This age group, currently exceeding 17 million individuals aged 60 and over, is projected to triple in the next 40 years [1,2]. As this population ages, they face increasing health challenges, including a high prevalence of chronic diseases such as obesity, diabetes, hypertension, and cardiovascular conditions [1]. In 2021, the most prevalent conditions among individuals aged 53 and over were hypertension (43.3%), diabetes (25.6%), and arthritis (10.7%). Additionally, 62.3% of this age group perceived their health status as fair to poor [3]. It has been demonstrated that comorbidities such as hypertension, obesity, and diabetes significantly increase their risk of hospitalization [4].

This scenario, combined with the health challenges faced by the rest of the population (individuals under 60 years old), predicts an unfavorable outlook for hospitalization services in Mexico. For instance, Rojas-Martínez et al. [5] estimate that approximately 10 million Mexicans could be at risk of developing diabetes or hypertension over the next decade. Furthermore, events such as the COVID-19 pandemic can overwhelm public health systems, further limiting the availability of timely and adequate medical care for older adults. This situation not only compromises the health and well-being of the population but also imposes a significant economic burden due to the high demand for hospital services.

Given these challenges, research on predictive models to anticipate hospitalizations in older adults becomes crucial. Predictive modeling offers a transformative approach to addressing these issues. Traditional methods, such as logistic regression, have been widely used to analyze risk factors for hospitalization and mortality during the COVID-19 pandemic [4]. However, these methods are limited in their ability to capture complex, nonlinear interactions between variables that characterize the health of older adults [6]. Advances in machine learning (ML) have demonstrated the potential to overcome these limitations, leveraging longitudinal and high-dimensional datasets to uncover intricate patterns in health determinants. This capability is particularly relevant in Mexico, where the availability of longitudinal data on older adults is relatively recent, marking an innovative step in research for this population.

In recent years, ML techniques have been applied to various health-related scenarios. Song et al. [7] demonstrated that random forest (RF) models achieved high performance in predicting hospitalizations among older adults with chronic diseases. Similarly, Kraus et al. [6] highlighted the utility of ML in predicting functional outcomes in older adults with orthopedic disabilities, emphasizing the need to integrate real-time clinical data.

The present study contributes to this growing body of research by applying ML techniques to the longitudinal Mexican Health and Aging Study (MHAS), a unique dataset that tracks the physical, emotional, and socioeconomic health of older adults in Mexico across multiple waves. The recent availability of these longitudinal data in Mexico makes this research particularly novel, as it allows for deeper and more precise analyses of their health and well-being over time. By employing the RF algorithm with rigorous validation strategies, including an adjusted version of nested cross-validation and temporal data partitioning, this work achieves robust predictive performance and offers insights into the importance of key variables influencing hospitalization risk.

The key innovations of this study are as follows:

- Highlighting the role of functional and socioeconomic factors, such as limitations in activities of daily living and educational level, as primary predictors of hospitalization, surpassing traditional clinical variables like hypertension, diabetes, and obesity.
- Improving the transparency and interpretability of ML models through variable importance analysis, addressing the common critique of ML methods as “black box” techniques.
- Applying advanced validation methodologies to ensure model generalizability and stability, as these are crucial to its real-world implementation in healthcare settings.

Despite significant advancements in ML applications, important gaps remain when it comes to understanding the hospitalization risk factors specific to older adults in Mexico, particularly with regard to integrating functional, clinical, and socioeconomic determinants. While prior studies have largely focused on clinical variables such as hypertension, diabetes, and obesity, our research adopts a more comprehensive approach by emphasizing the importance of functional and socioeconomic factors.

This study innovates by applying a random forest algorithm to the longitudinal MHAS dataset to predict hospitalizations among older adults, an approach that, to our knowledge, has not been previously implemented for the Mexican elderly population. Our methodology combines rigorous validation strategies with interpretability through variable importance analysis. By including functional limitations and educational level as key predictors, this study challenges traditional assumptions that prioritize clinical factors, offering novel insights for targeted public health interventions.

## 2. Related Work

Several studies have explored the use of predictive models in healthcare, targeting diverse prediction objectives and application scenarios.

Kraus et al. [6] developed an ML-based approach to predict the Time-Up-and-Go (TUG) test in older adults with orthopedic disabilities. Using 67 multifactorial parameters unrelated to mobility and six feature selection algorithms during preprocessing, they trained four ML models: a generalized linear model, support vector machine (SVM), random forest (RF), and XGBoost. Five-fold internal cross-validation was applied, with an 80/20 split for training and validation. RF algorithm demonstrated the best performance, highlighting its effectiveness for risk stratification in clinical settings. However, the study emphasized the need for real-time clinical data and larger datasets to improve prediction accuracy.

Similarly, Song et al. [7] examined hospitalization risk in patients over 65 years old with chronic diseases and COVID-19. Their study employed four ML algorithms: regularized logistic regression, SVM, RF, and neural networks. The RF model achieved the best performance, with an area under the curve (AUC) of 0.83, due to its ability to capture complex relationships between variables and avoid overfitting by combining multiple decision trees. This study underscores the potential of ML to identify high-risk individuals and enable early interventions to prevent complications.

In another application, Taloba et al. [8] compared linear regression, Naïve Bayes, and RF models to predict hospitalization and healthcare costs based on risk factors such as body mass index (BMI) and demographic characteristics. The linear regression model achieved the best performance, with a predictive accuracy of 97.89%. However, their exclusive reliance on the accuracy metric is a notable limitation, as sensitivity and specificity are crucial for imbalanced datasets in healthcare.

Kandel et al. [9] investigated hospitalization prediction in skilled nursing facilities using the LightGBM algorithm, which is known for its high computational efficiency when dealing with large datasets. They identified key risk factors, including comorbidities and levels of dependency in daily activities. LightGBM achieved strong performance using metrics such as F1-score, sensitivity, and positive predictive value. However, the study highlighted challenges with hyperparameter tuning and overfitting when using LightGBM, suggesting that simpler methods like RF may offer a better balance between performance and clinical applicability.

The study by Friesner et al. [10] focused on predicting unplanned hospitalizations during chemotherapy and radiotherapy using physical activity data collected through wearable devices. The analysis included 214 patients with various types of cancer. The results showed that the evaluated models had very similar outcomes, with regression model achieving an area under the curve (AUC) of 0.83, followed by the neural network with an AUC of 0.80 and the RF with an AUC of 0.76. The study demonstrated the importance of integrating physical activity data into models to improve the identification of patients at risk of complications during oncological treatments and enhance predictive accuracy. For more insights, refer to the work of Durán-Vega et al. [11], which emphasizes the significance of wearables in health monitoring. However, the study has some limitations: a short data collection period, academic setting bias, and a lack of complex clinical data, which may have affected the predictive capacity of the models. These insights highlight the need for multidimensional data in predictive models, particularly for older adult populations.

Ermak et al. [12] evaluated ten ML models for predicting hospitalizations in patients with coronary artery disease, identifying CatBoost as the top performer (AUC: 0.875) due to its ability to handle imbalanced data and categorical variables. However, this study has several significant limitations. The selection of the best model was based solely on the area under the receiver operating characteristic curve (AUROC), and the model was complex, utilizing techniques such as RandomGridSearch and multiple iterations for hyperparameter optimization. This, combined with the inherent complexity of CatBoost and its lower interpretability, presents substantial challenges for its adoption in clinical settings, where simplicity and speed are essential [13].

A systematic review by Amanollahi et al. [14] examined ML models used to predict relapses, hospitalizations, and suicides in bipolar disorder patients. The review, which included eighteen studies and over 30,000 patients, identified RF, SVM, and logistic regression as the most used models. They emphasize the robustness of RF in handling imbalanced and noisy data, reducing prediction error in clinical contexts. The authors also highlighted the significance of using nested cross-validation, a technique rarely employed in the reviewed studies, and emphasized the necessity of using metrics such as sensitivity and specificity to predict adverse clinical events.

In Mexico, predictive models for older adults remain scarce, particularly those leveraging longitudinal data. Logistic regression has traditionally been the preferred method due to its ease of implementation and interpretability. For example, Carrillo-Vega et al. [4] identified hypertension, obesity, and diabetes as key risk factors for hospitalization and mortality in COVID-19 patients. Their findings revealed that men had a 1.54 times higher risk of hospitalization compared to women (*p*-value < 0.001, 95% Confidence Interval: 1.37–1.74). Additionally, individuals aged 75 years or older had a significantly higher risk of hospitalization compared to those aged 49 years, with an odds ratio of 3.84. However, the linearity assumptions of logistic regression limits its ability to capture the complex patterns in health data [15], underscoring the need for ML approaches.

To address this gap, our study leverages the Mexican Health and Aging Study (MHAS) [16], a comprehensive longitudinal dataset on the health, socioeconomic status, and well-being of older adults in Mexico. Using random forest, a model well suited to the complexity of health data, this research develops a robust framework for hospitalization risk prediction. The novelty of this approach lies not only in the application of machine learning to a Mexican longitudinal dataset but also in the methodological rigor applied—employing techniques such as nested cross-validation to ensure reliable performance and generalizability. By identifying key risk factors and patterns, the results of this study can inform targeted interventions, improve resource allocation, and support equitable healthcare planning for Mexico’s aging population.

## 3. Materials and Methods

The objective of this study is to develop a predictive model to identify future hospitalizations in older adults. To achieve this, a prediction strategy was designed using the RF algorithm and the MHAS dataset [16]. The MHAS dataset comes from a national longitudinal study. We are using information from 2012, 2015, and 2018. MHAS includes the socioeconomic, health, and lifestyle information of individuals 50 years and older, residing in various states in Mexico.

The RF algorithm was selected for this study due to its ability to handle datasets with multiple predictor variables and its robustness against overfitting, a crucial aspect in medical applications [14]. Additionally, this algorithm is particularly suitable for processing binary, categorical, and ordinal variables, which constitute the majority of our dataset. Not assuming linear relationships between variables allows us to capture more complex interactions among predictive factors, regardless of the variables used [7,17]. Therefore, RF is essential for predicting events such as hospitalizations, where the factors influencing the likelihood of hospitalization are complex and often do not follow linear patterns. Factors such as age, health status, access to medical services, and socioeconomic conditions interact in ways that cannot always be represented by simple cause-and-effect relationships, making RF the best option for this analysis.

The mathematical model that describes the internal workings of the RF algorithm is presented below. The algorithm’s prediction is based on the following formulations [18,19]:

1. Model Definition

RF is an ensemble learning method that functions by constructing multiple decision trees during training and aggregating their outputs (e.g., majority vote for classification) to improve overall predictive accuracy and reduce overfitting. Formally, RF combines individual decision trees $\left\{T_{1},T_{2},\ldots ,T_{B}\right\}$, where B represents the total number of trees. Their prediction is based on majority voting:

(1) $$ŷ=mode\left\{T_{1}\left(x\right),T_{2}\left(x\right),\ldots ,T_{k}\left(x\right)\right\},$$

2. Building an Individual Tree

For each tree, the following stages are utilized:

Bootstrap Aggregation (Bagging): A random sample $D_{b}$ is generated with replacement from the original D dataset.

RF selection: At each node of the tree, a random subset of m features is selected (usually $m=\sqrt{p}$, where p is the total number of features).

Optimal splitting: The tree is split by selecting the feature j and the split point s that maximizes the gain criterion. For classification tasks, RF leverages the Gini impurity to measure node splits within individual trees. The Gini impurity $G\left(j,s\right)$ is computed for each possible split at node j and cut point s, and the algorithm selects the split that minimizes the Gini impurity:

(2) $$G\left(j,s\right)=1-\sum_{c}{\left(p_{c}\right)}^{2},$$

where $p_{c}$ represents the proportion of samples at node j that belong to class c. The summation is performed over all possible classes c.

This Gini impurity value is used to assess how impure or disordered a node is. The algorithm then selects the split j and split point s that minimize this impurity, thereby making more accurate decisions when building the tree.

3. Bagging Mathematics

Bagging reduces the variance of the model. If an individual tree has a variance σ^2^, then the RF has the following variance:

(3) $$Var\left(ŷ\right)=\frac{\sigma^{2}}{B}+\left(1-\rho \right)\sigma^{2},$$

where ρ is the correlation between the trees. Randomization in feature selection decreases ρ, reducing the overall variance.

4. Importance of Variables

The importance of a variable $X_{j}$ is measured as follows:

Average impurity reduction:

(4) $$Importance\left(X_{j}\right)=\sum_{t\in T}∆I\left(t,X_{j}\right),$$

where $∆I\left(t,X_{j}\right)$ is the gain in impurity when using $X_{j}$ to split node t.

Permutation of values measures how much the model’s performance degrades when permuting the values of $X_{j}$.

This formal framework underpins the construction of RF models used in this study. These properties enable the algorithm not only to deliver accurate predictions but also to rank predictors based on their contribution. This is a crucial feature, as the study aims to identify the key factors influencing the risk of hospitalization. In the next section, we provide a general review of the dataset used for this study, outlining its structure and key variables that contribute to the prediction model.

### 3.1. General Review of the Dataset

The dataset used in this study is derived from the Mexican Health and Aging Study (MHAS), a national longitudinal study of adults aged 50 and older, which was partially funded by the National Institutes of Health (NIA), the National Institute on Aging (NIH), and the Mexican National Institute of Statistics and Geography (INEGI). Data collection waves were conducted in 2001, 2003, 2012, 2015, 2018, and 2021. The study’s protocols and instruments are highly comparable to those used in the U.S. Health and Retirement Study (HRS), ensuring cross-national comparability. The study included private households with at least one resident aged 50 or older, randomly selecting one eligible participant per household. If the selected participant was married or cohabiting, their spouse was also recruited, regardless of age [16].

For the purposes of this research, the MHAS dataset underwent a preprocessing phase specifically designed and executed to meet the study’s requirements. This phase included the removal of empty fields, handling incomplete data through deletion, selection of individuals aged 50 and older, and the appropriate encoding of each input variable (e.g., binary and ordinal categorical encoding). After this preprocessing stage, the final dataset comprised 30,603 rows and 17 columns. A description of these columns is provided below:

- Fourteen columns correspond to the predictor variables: sex, age, diabetes, stroke, educational level (years of education completed), place of residence (urban or rural), living with someone, body mass index, hypertension, number of limitations in basic activities of daily living (n-abvd, for its acronym in Spanish), falls, access to public health services, smoking history, and current alcohol consumption;
- Two columns represent the individual’s ID and the year in which the information was collected;
- Finally, there is a column for the target variable: hospitalization in the last 12 months. The MHAS dataset shows a marked imbalance in this variable (with hospitalization rates of 9.9% in 2012, 12.8% in 2015 and 15.6% in 2018), which must be considered in the machine learning model design to avoid biased results.

A descriptive summary of the dataset is presented in Table 1. The data are organized by year and expressed as percentages for each variable used in the machine learning model.

The analysis focused on data from the years 2012, 2015, and 2018 to maintain a consistent temporal pattern. Earlier waves from 2001 and 2003 were excluded due to the significant gap between them and subsequent years, which could disrupt the temporal continuity necessary for robust longitudinal analysis. Additionally, the 2021 wave was excluded because the COVID-19 pandemic represents a different scenario for hospitalization. By focusing on these three years, the model was able to better capture the progression of hospitalization risks over time.

### 3.2. Study Evaluation Metrics

The primary objective of the experiments was to identify the model that optimizes the balance between sensitivity and specificity, with the aim of minimizing both false positives and false negatives—a critical factor in medical applications such as hospitalization prediction [14]. Therefore, the performance of the model was evaluated using the following metrics:

- Kappa coefficient: a statistic that measures the agreement between predictions and observations adjusted for the possibility that predictions could be made by chance;
- Sensitivity: also known as “recall”, this metric measures the model’s ability to correctly identify positive cases (hospitalizations);
- Specificity: this metric assesses the model’s ability to correctly identify true negative cases (non-hospitalizations), helping to reduce the number of false positives that could lead to an unnecessary strain on hospital resources.

Due to the significant imbalance in the dataset—the percentage of hospitalizations is relatively low (comprising only 12.8% of the 30,603 records)—the accuracy metric was excluded from the analysis. In situations where there is an imbalance in the target variable, accuracy tends to favor the majority class (non-hospitalizations), which does not adequately reflect the model’s performance in identifying the minority class. Consequently, accuracy is not an appropriate metric in this context, as it could lead to misleading interpretations and underestimate the model’s performance in predicting the minority class (hospitalizations). Instead, we focused on sensitivity, specificity, and the kappa coefficient, which more accurately captures the model’s behavior in the face of data imbalance and aligns with the study’s goal of correctly identifying both true positive and true negative cases.

### 3.3. Machine Learning Model Design

**Figure 1:**
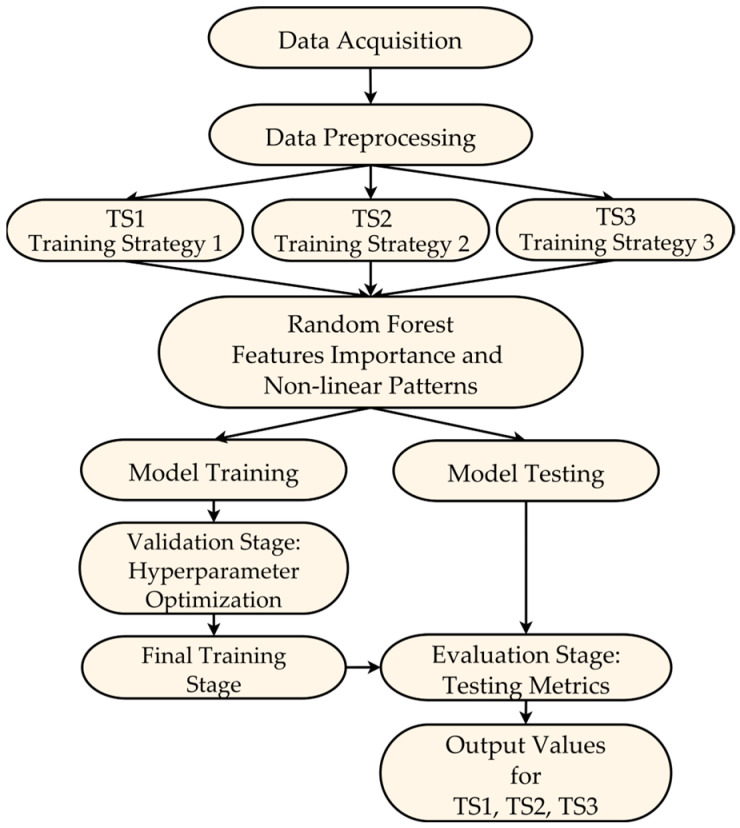
Diagram of machine learning model design.

This section describes the design of the machine learning model used to predict hospitalizations in older adults, specifically the use of the RF algorithm and the temporal data partitioning strategies for training and testing. As illustrated in Figure 1, the design process is structured into three main aspects: the data partitioning strategy, the model training and testing process, and the final evaluation on independent test data. A detailed description of each aspect will follow, where we will explain the design decisions and procedures that underpin these stages.

#### 3.3.1. Data Partitioning Design for Training and Testing

To predict hospitalizations, a machine learning model based on the RF algorithm is proposed, using three temporal strategies for data partitioning for training and testing of the model (ST1, ST2 and ST3), as shown in Figure 1. Data partitions for training and testing were performed using information from different years. The configuration of the three temporal data partitioning strategies was as follows:

- Strategy 1 (ST1): The model is trained and evaluated considering data from the three periods (2012, 2015 and 2018) jointly for each individual.
- Strategy 2 (ST2): The model uses only 2012 data to predict hospitalization events that occurred in 2015 and 2018.
- Strategy 3 (ST3): The model predicts hospitalization events in 2018, using data collected in 2012 and 2015 as part of the training set.

Figure 1 presents the complete modeling workflow, encompassing the stages from data acquisition and preparation to the training and testing phases of the ML model. This diagram highlights the different test data partitioning strategies (TS1, TS2, TS3) employed, which were crucial for evaluating the model’s generalization across different configurations. Furthermore, the proposed model design and testing approach provides a comprehensive view of how hospitalizations evolve over time.

#### 3.3.2. Model Training and Testing

The predictive model was trained using advanced techniques to ensure robustness and minimize the risk of overfitting. The RF algorithm employed bagging and random feature selection during the training of individual trees. Bagging involved generating bootstrap subsets of the data, which helped reduce model variance, while random feature selection at each tree node decreased correlations between trees, thereby optimizing the model’s predictive capacity. Additionally, the RF algorithm utilized the Gini criterion to evaluate split points, selecting those that maximized node purity gain, thus enhancing the model’s decision-making accuracy. Furthermore, to assess variable importance, two complementary approaches were applied: (1) mean impurity reduction, which measures the contribution of each variable based on its impact on the split nodes during model training, and (2) permutation of values, which estimates the degradation in model performance by randomly altering the values of a specific variable after the model has been trained. The latter approach evaluates a variable’s importance by observing how much the model depends on it for making accurate predictions. These analyses were crucial for identifying key factors associated with hospitalization risk.

During the model training and testing phase, a modified nested cross-validation scheme was proposed, which was tailored to the specific characteristics of the available data and the goal of predicting future hospitalizations in older adults. This scheme consists of two main levels: internal validation, external validation, followed by a final evaluation of independent test data (Figure 1):

1. Inner validation (hyperparameter optimization): In the internal validation process, cross-validation is used with the training set to perform the model’s hyperparameter optimization. This stage includes the following steps:
  - Division of the internal training set: The dataset defined as the internal training set (e.g., 90% of the external training set in the 90–10% outer-validation split) is further subdivided into k-folds (or subsets, with *k* = 10) for cross-validation. In each iteration, *k* − 1 partitions are used to train the model, while the remaining partition is used for validation. This process is repeated *k* times, ensuring that each subset serves as a validation set once;
  - Hyperparameter tuning: In each iteration, different hyperparameter configurations, such as the number of trees in the random forest and the maximum depth of the trees, are evaluated based on the metrics obtained in each iteration. At the end of this process, the hyperparameters that maximized the model’s performance in the internal validations are selected.
2. Outer validation (Generalization Assessment): The main objective of external validation is to measure the model’s performance in response to entirely new data that were not used during the optimization process. This stage consists of the following steps:
  - Initial data splitting: The entire dataset is split into training and testing sets according to the partitioning strategy used (ST1, ST2, and ST3). For example, in ST3, the data from 2012 and 2015 formed the training set, while the 2018 data were reserved as the final or independent testing set;
  - Subdivision of the external training set: The training partition (2012 and 2015 data in the case of ST3) is divided into 2 new subsets: internal training and internal testing. This subdivision is performed according to specific proportions based on the following proposal: 0.1 (10–90%), 0.2 (20–80%), 0.3 (30–70%), and 0.4 (40–60%). For example, in the 0.1 proportion, the internal training set would contain 90% of the data, and the internal testing set would contain the remaining 10%;
  - Retraining with optimal hyperparameters: Once the optimal hyperparameters have been selected in the internal validation stage, the model is retrained using the entire internal training set. This ensures that the model will leverage the maximum amount of data before proceeding to evaluation;
  - Evaluation on the external testing set: The retrained model is evaluated on the internal testing set (e.g., 10% of the data defined as part of the outer validation) to measure its performance on unseen data.
3. Final Model Evaluation: After completing the inner validation and outer validation stages, a final independent evaluation was conducted using the test set reserved at the beginning of the data partitioning strategy (2018 data in the case of ST3). These data were not involved in any previous training or optimization stages. This step allowed reporting of the final metrics and assessing the model’s generalization capacity for predicting future hospitalizations.

In summary, this modified nested cross-validation approach integrates internal hyperparameter optimization with external validation and culminates in a final evaluation of independent test data, ensuring robust and realistic performance estimates. This design ensures the integrity and independence of the training, validation, and test sets, and accurately measures the model’s generalization capability.

#### 3.3.3. Relevance of the Proposed Design Strategy

The design of the internal and external cross-validation stages implemented in this study effectively addresses two key objectives: preventing model overfitting to the training data and ensuring that the performance measured on the test data accurately reflects the model’s generalization capability. This approach ensures that hyperparameters are adjusted only within the training set, while evaluation is conducted on completely independent data in each phase, strengthening the reliability of the obtained results and the model’s ability to predict unseen future data.

Additionally, the inclusion of different test partitioning proportions (0.1, 0.2, 0.3, and 0.4) provides a more robust view of the model’s performance under various data configurations. This is especially relevant in scenarios with imbalanced data, as is the case with hospitalization prediction, since it allows for the assessment of how the model handles variability in data distribution.

In conclusion, this design proposal not only enabled the evaluation of model performance in multiple scenarios but also appropriately integrated the longitudinal nature of the data, offering a more robust and representative view of its performance. Furthermore, this strategy ensures the independence of training, validation, and test sets, preventing the improper use of test data and ensuring that the model has a strong capacity to generalize new data. This design fulfills one of the main objectives outlined in this work: to provide a solid and realistic evaluation of the model’s performance in the context of predicting hospitalizations for older adults based on the selected input variables.

### 3.4. Case Studies

The main objective of these experiments is to evaluate the performance of the model in several data partitioning scenarios (ST1, ST2, and ST3) to identify the most effective combination for predicting hospitalizations in people aged 50 years or older. The final selection of the model and its partitioning strategy was based exclusively on the evaluation metrics described above, aiming for an optimal balance between sensitivity and specificity. In this context, two case studies were designed to assess the effectiveness of the model:

- Case 1 (variables without interaction): uses 14 predictor variables, as previously mentioned, along with the individual’s ID and the year the data were collected;
- Case 2 (variables with interaction): includes the 14 original predictor variables and all possible first-order interactions, meaning combinations between each pair of the 14 input variables.

The inclusion of variable interactions aims to capture additional relationships between predictive factors that could influence hospitalizations. This approach enriches the information provided to the machine learning model, allowing for an exploration of whether these interactions enhance predictive performance.

These two case studies enable a comprehensive evaluation of the model under different data configurations, providing a holistic view of their performance. This approach allows for the identification of the model with the best data partitioning strategy, thereby ensuring robust and reliable application in real-world settings.

## 4. Results

This section presents the main findings of this study on predicting hospitalizations among older adults using a machine learning model based on the RF algorithm. It begins with a detailed analysis of the importance of predictive variables, enabling the identification of key factors influencing the model’s ability to forecast hospitalizations. Subsequently, the overall performance of the model is evaluated using the three data partitioning strategies, followed by an analysis to determine the best trade-off between sensitivity and specificity.

### 4.1. Importance of Predictor Variables

**Figure 2:**
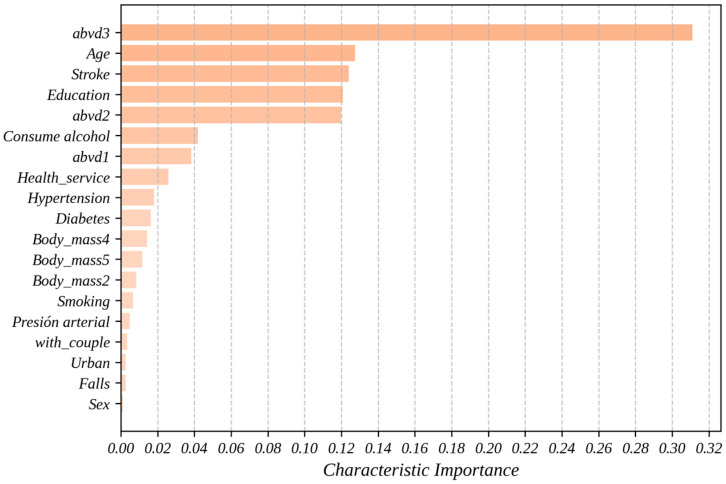
Importance of predictor variables in predicting hospitalizations in older adults. Values on the X-axis have been normalized between 0 and 1.

Figure 2 shows the importance of the predictor variables obtained through the predictive model using the RF algorithm. Beyond its ability to make accurate predictions, machine learning allows for the identification of complex and nonlinear patterns in the data, providing a deeper understanding of the factors influencing hospitalization in older adults. In this context, the importance of a variable represents its contribution to the model’s ability to predict the target variable. The analysis was conducted using two complementary approaches: mean decrease in impurity and permutation importance, as previously described in the Methodology section of this paper. These methods allow for the evaluation of each variable’s role in the model’s performance from different perspectives, facilitating a more robust and well-founded interpretation.

With a value of 0.3110, abvd3 (a score of 3 in abvd, indicating three limitations in basic activities of daily living) is the variable which makes the greatest contribution to the model, significantly surpassing the rest of the variables used. This means that this characteristic explains approximately 31.1% of the total predictive capacity of the proposed model. This confirms that difficulties in daily activities are a critical factor for predicting hospitalizations and should be considered in health prevention designs.

Another finding from Figure 2 is that the variables age, stroke (history of cerebrovascular accident), education (individuals with 6 or more years of education), and abvd2 demonstrated relevant contributions, positioning themselves near the middle of the variable importance graph in predicting hospitalizations. In this study, age ranks second with a value of 0.1275 (12.75% influence on the predictive model), supporting its direct relationship with the probability of hospitalization due to physiological aging and the accumulation of comorbidities. With the importance of 0.1240, the history of cerebrovascular accident showed a significant contribution, aligning with previous studies that associate this condition with higher risks of hospitalization.

The variables education and abvd2 also proved to be relevant, with values of 0.1208 and 0.1198, respectively, suggesting that educational level and additional functional difficulties play an important role in prediction. The relevance of the education variable, although surprising, suggests its influence on the degree of hospitalization among adults and highlights the importance of considering socioeconomic factors in the prediction of health events. In fact, this may suggest that individuals with higher levels of education may have greater health knowledge, tend to adopt healthier lifestyles, and have access to resources that allow them to better care for their health and prevent diseases.

Finally, although factors such as consumption of alcohol (0.0420), abvd1 (0.0384), Health_service (0.0259), hypertension (0.0181), diabetes (0.0162), and Body_mass4 (0.0142) had lower contributions, they remain important elements in the analysis. The body mass index was evaluated across five categories, ranging from underweight (Body_mass1) to morbid obesity (Body_mass5). Additionally, variables related to lifestyle habits (e.g., smoking, 0.0067), socioeconomic conditions (e.g., with_couple, 0.0048; Urban, 0.0035), and gender (Sex, 0.0026) showed lower values (<0.02), but they are still relevant. Their contribution, although modest, could interact with other variables or have a cumulative effect on the prediction.

### 4.2. Overall Performance of the Model

**Figure 3:**
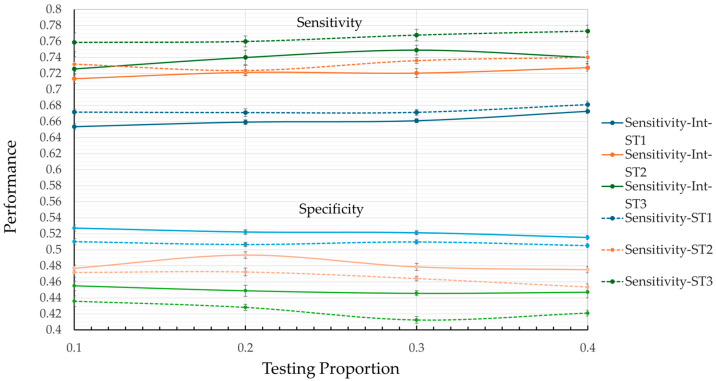
Model’s performance in terms of sensitivity and specificity: under ST1, ST2, and ST3 and in the two case studies.

**Figure 4:**
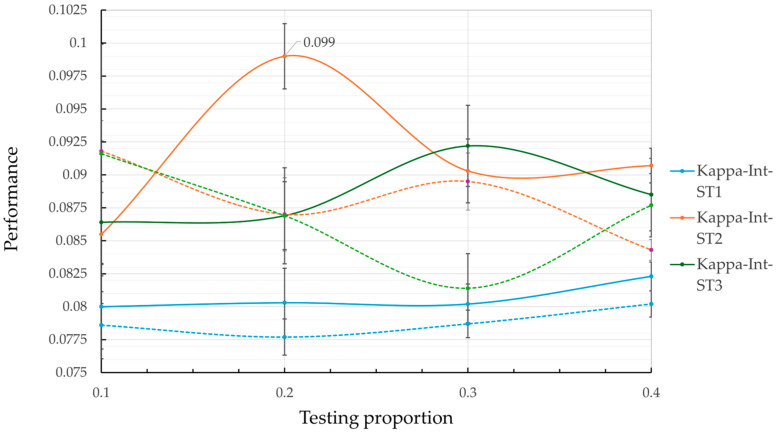
Result of the kappa coefficient for ST1, ST2, and ST3 in the two case studies.

Table 2 and Figure 3 and Figure 4 present the average results and standard error values for each metric, obtained by the model for each data partitioning strategy (ST1, ST2, and ST3), when applying the RF algorithm to the MHAS dataset with different test proportions. These results not only provide insight into the models’ predictive performance but also reflect how effectively the selected predictor variables contribute to their success. The results vary depending on the presence or absence of variable interaction and the test proportion, emphasizing the importance of accounting for interactions among predictors in complex health scenarios.

In general, the low standard error values across all results and configurations demonstrate high precision and stability in the metric estimates, highlighting the robustness of the predictor variables identified during the feature selection process. Moreover, the lack of overlap in the error intervals between results—particularly for sensitivity and specificity—demonstrates that the observed performance differences are statistically significant, further validating the predictive value of the variables included.

Although the overall results exhibit consistent reliability across configurations, the results with variable interaction stand out by achieving lower error margins on average. It is worth noting that this behavior is more present in the specificity metrics and the kappa coefficient. This enhanced performance highlights the importance of leveraging both individual predictor contributions and their interactions, enabling a more nuanced and accurate modeling of complex phenomena like hospitalization risk in older adults. The results confirm the capacity of ML approaches to not only predict outcomes but also to uncover underlying patterns and relationships in health-related data. In terms of the average performance values of the metrics, Figure 3 shows that, in general, the model tends to perform better in terms of sensitivity than specificity, regardless of the test ratio. Furthermore, the model without interaction (dotted lines) excels in the sensitivity category, with ST3 reaching its highest value (0.7729) at a test ratio of 0.4. However, this result is accompanied by poor performance in terms of specificity (0.4124) and the kappa coefficient (0.0814) (see Table 2), which may lead to an increase in false positives.

From Figure 3 and Figure 4, it is observed that ST1 achieves the highest values for specificity, with a maximum value of 0.5271 at a test ratio of 0.1. However, its low performance in terms of sensitivity (0.6537) and kappa coefficient (0.08) suggests a significant risk of failing to correctly identify hospitalization cases, which is a critical aspect of medical prediction.

An important observation from Figure 3 and Figure 4 is that ST2 is the only model that presents balanced results across the metrics studied. In sensitivity, ST2 remains significantly greater than ST1 and approaches the performance of ST3 across all test ratios. Comparing the best values, ST1 reaches 0.6812 while ST2 achieves 0.7402, representing an 8.7% increase in sensitivity. The percentage difference between ST2 (0.7402) and ST3 (0.7729) is 4.2% in the same metric. A similar pattern is observed in the specificity metric, where ST2 outperforms ST3 and approaches ST1.

Regarding the kappa coefficient metric (Table 2 and Figure 4), results that include variable interaction consistently outperform those without interaction across almost all test ratios. The best performance in the kappa coefficient was achieved by ST2, which reached a value of 0.099 at a test ratio of 0.2. The typical error of ±0.0248 suggests that this result is statistically significant, with no overlap in error intervals compared to other strategies. This indicates that ST2 presents better agreement between predictions and actual observations, highlighting its effectiveness compared to the other evaluated strategies.

### 4.3. Machine Learning Model Selection

In the context of predicting hospitalization, it is preferable to balance the performance metrics of ML models rather than focusing exclusively on maximizing a single metric. A balance between sensitivity and specificity is crucial to avoid biases that may lead to the overestimation or underestimation of hospitalizations.

**Figure 5:**
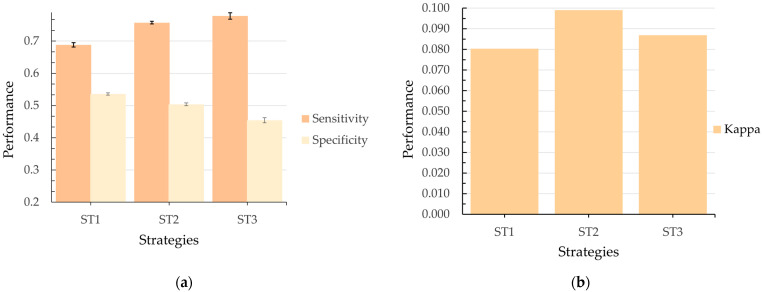
(**a**) Model performance in sensitivity and specificity for ST1, ST2, and ST3 with variable interaction and a test ratio of 0.2; (**b**) kappa coefficient performance for ST1, ST2, and ST3 under the same configuration.

Among the evaluated strategies, the model with ST2, incorporating variable interaction and a test ratio of 0.2, stands out because it offers an optimal balance. This strategy achieved a sensitivity of 0.7215 and a specificity of 0.4935 (Figure 5a), effectively minimizing both false negatives and false positives; minimizing these is crucial for accurate medical prediction. The standard errors of ±0.0038 for sensitivity and ±0.0039 for specificity confirm that the differences between the results are statistically significant. Additionally, the model with ST2 achieved the highest value in the kappa coefficient metric (0.099), as shown in Figure 5b, reflecting greater agreement between the predictions and the actual observations compared to the other strategies analyzed. Consequently, ST2 efficiently balances the identification of positive and negative cases, thus meeting the study’s objectives. Therefore, ST2, with variable interaction and a 20% test ratio, is selected as the optimal model for predicting hospitalization in older adults in Mexico. Moreover, based on ST2’s results, we can conclude that the model’s practical viability in medical prediction is highlighted, contributing to better anticipation of hospital demand in health services.

## 5. Discussion and Future Research Directions

The findings of this study highlight the potential of machine learning models to predict hospitalizations among older adults in Mexico, particularly when using longitudinal data. While the model with the ST2 achieved a sensitivity of 0.7215 (±0.0038), the relatively lower specificity (0.4935 ± 0.0039) reflects the inherent trade-off in predictive modeling: prioritizing sensitivity to avoid missing cases often comes at the expense of increased false positives. This trade-off is consistent with previous studies on health predictions in older populations [9]. Importantly, optimizing sensitivity is critical in contexts like Mexico, where early identification of high-risk individuals can guide preventive interventions and resource planning. However, future research should aim to improve this balance, possibly through stratified approaches or by incorporating clinical biomarkers and real-time health monitoring.

A major contribution of this study is the analysis of predictor variable importance (Figure 2), which deepens the understanding of factors driving hospitalizations in older adults. The abvd3 variable, representing limitations in three basic activities of daily living, emerged as the most influential predictor, contributing 31.1% of the model’s performance. This finding highlights the pivotal role of functional limitations in hospitalization risk and underscores their importance for public health interventions targeting older adults. Additionally, other key predictors included age, history of cerebrovascular accidents, educational attainment, and limitations in two basic activities of daily living (abvd2).

The identification of educational attainment (12.08%) as a significant predictor is particularly noteworthy, suggesting indirect pathways through which education influences hospitalization risk. These pathways may include better health literacy, healthier lifestyles, and improved healthcare access. This result aligns with and expands previous research, emphasizing the need for further exploration of socioeconomic determinants in health outcomes.

Interestingly, some traditionally prominent risk factors, such as hypertension (0.0181), diabetes (0.0162), and obesity categories (e.g., Body_mass4, 0.0142), had relatively lower contributions compared to functional and socioeconomic variables. This challenges conventional assumptions and suggests that these factors might act synergistically with others or could be less predictive in heterogeneous populations like the one studied here. These findings indicate the need for tailored approaches in predictive modeling that account for the interplay between clinical, functional, and socioeconomic predictors.

The integration of variable importance analysis enhances the interpretability of machine learning models, addressing a common critique of “black box” methods. By providing insights into the relative contributions of predictors, this study strengthens the practical applicability of machine learning for public health decision-making. Such interpretability is crucial for guiding tailored interventions aimed at reducing hospitalization risk among older adults.

### 5.1. Limitations and Future Directions

Despite its contributions, this study has some limitations. First, the reliance on self-reported data, which are commonly utilized in large-scale surveys like MHAS, may introduce potential biases. Furthermore, incorporating clinical biomarkers, such as glucose or cholesterol levels, into future datasets could improve our model’s predictions. Similarly, integrating real-time data from wearable devices may improve the timeliness and precision of predictions, as demonstrated in studies predicting unplanned hospitalizations during concurrent chemotherapy and radiotherapy [10].

The moderate performance of the model may relate to the dataset’s heterogeneity, which includes individuals with varying health conditions and an imbalance between hospitalization cases (12.8%) and non-hospitalized cases (87.2%). This imbalance likely impacted sensitivity and specificity values. Additionally, our research employed a rigorous methodology, including modified nested cross-validation, temporal data partitioning strategies, and comparisons with and without variable interactions. These methods, combined with the complexity of predicting hospitalizations in a diverse population, may have resulted in our proposed model exhibiting a more conservative performance compared to previous studies. Song et al. [7], for example, achieved more favorable results in predicting hospitalizations in patients with specific comorbidities by working with more homogeneous datasets using the RF algorithm. Nevertheless, while the methodological rigor of our approach may have exposed some limitations in the performance metrics, it also provides a clearer view of the model’s actual performance under more complex conditions. Future studies should explore stratified modeling approaches, as proposed by some previous studies [4,12,14], focusing on specific subpopulations (e.g., individuals with chronic conditions or comorbidities) to reduce heterogeneity and improve predictive accuracy.

Additionally, implementing advanced techniques for handling missing data, beyond record elimination, could preserve valuable information and improve model performance. While the random forest algorithm demonstrated effectiveness, future studies could benefit from testing ensemble methods like Gradient Boosting Machines (GBMs) or neural networks to enhance predictive capacity further.

### 5.2. Border Implications

The implications of this study extend beyond the Mexican context. The identified predictors, such as functional limitations, socioeconomic factors, and comorbidities, are common across aging populations globally. This model can be adapted to predict hospitalizations in other regions, aiding in the proactive allocation of healthcare resources.

By combining robust predictive methodologies with interpretable insights, this research represents a significant step toward the integration of machine learning in public health planning. Future efforts should continue to refine this model while expanding their applications to diverse healthcare systems facing similar challenges with aging populations. This expansion not only offers the potential to optimize healthcare resource planning but also provides a valuable opportunity to deepen the understanding of the health conditions of older adults in Mexico and similar contexts.

In summary, this study demonstrates the value of machine learning not only for accurate predictions but also for uncovering critical factors influencing hospitalizations. The insights derived from variable importance analysis pave the way for more targeted interventions, while the methodological rigor provides a blueprint for future research. Addressing the identified limitations and pursuing the suggested directions will further enhance the utility and impact of predictive models in healthcare systems worldwide.

## 6. Conclusions

Machine learning techniques, such as the random forest algorithm, have proven effective in predicting hospitalizations among older adults by addressing the complexity of health-related factors. In this study, the model applied to the longitudinal Mexican Health and Aging Study (MHAS) dataset achieved a good balance between sensitivity (0.7215) and specificity (0.4935) under the ST2 partition strategy. Internal validation confirmed its stability and generalizability, reflecting the trade-off between minimizing missed cases and controlling false positives, which is crucial in preventive healthcare where early identification is critical.

The variable importance analysis revealed that limitations in basic activities of daily living (abvd3) were the most significant predictor, with a 31.1% contribution to the model’s performance. Other key factors included age (12.75%), history of stroke (12.40%), educational level (12.08%), and difficulties in two basic activities of daily living (abvd2, 11.98%). The identification of education level as a relevant factor highlights the need to consider socioeconomic determinants in public health strategies.

Interestingly, clinical factors like hypertension, diabetes, and obesity had smaller contributions, challenging traditional assumptions and suggesting a complex interaction with other predictors. These findings emphasize the importance of personalized approaches that integrate functional, clinical, and socioeconomic factors.

By combining robust predictive performance with interpretability, this study addresses common critiques of machine learning as “black box” methods. The insights into variable importance not only improve transparency but also enable targeted public health interventions, particularly for populations with functional limitations and socioeconomic vulnerabilities.

Despite its moderate performance, the model benefited from a rigorous methodology, including adjusted version of nested cross-validation and temporal partitioning strategies. However, limitations such as reliance on self-reported data and dataset heterogeneity remain. Future research should address these issues by exploring stratified models for specific subpopulations, incorporating clinical biomarkers and real-time health monitoring, and testing additional machine learning algorithms, such as Gradient Boosting Machines or neural networks, to further improve performance.

In conclusion, this research demonstrates the utility of machine learning for both prediction and understanding of hospitalization risk factors. The implementation of this type of predictive model is essential for addressing the challenges posed by population aging in Mexico, as well as for optimizing resource use in a context where demand exceeds the healthcare system’s capacity. Future research should focus on integrating subgroups of older adults with specific comorbidities to enrich the model and improve its predictive capacity.

## Figures and Tables

**Table 1:** Descriptive table of preprocessed dataset from the original Mexican Health and Aging Study (MHAS).

	2012	2015	2018
Records	10,201	10,201	10,201
Hospitalized	9.9	12.8	15.6
50 ≥ Age < 60	34.68	23.99	13.11
Age ≥ 60	65.32	76.00	86.88
0 ≤ Education ≤ 6	71.54	71.54	71.54
6 < Education ≤ 12	20.24	20.24	20.24
12 < Education ≤ 22	8.21	8.21	8.21
Women	58.2	58.1	58.1
Urban (≥100 thousand)	55.5	54.7	54.7
Lives with a Companion	70.14	66.06	62.04
With Diabetes	22.1	25.0	27.6
With Hypertension	43.6	48.5	50.3
With Stroke	1.8	2.1	3.1
With Falls	39.7	44.6	44.5
With Access to Health Services	88.1	91.8	92.5
With Difficulty in 1 + ADL	11.8	14.9	17.4
Underweight	1.0	1.3	1.6
Normal	29.4	31.0	32.2
Overweight	44.0	43.8	42.9
Obesity I	24.0	22.4	21.9
Obesity II	1.6	1.6	1.4
Has Smoked	36.1	39.4	37.2
Has Consumed Alcohol	23.6	23.0	23.5

Values are expressed as a percentage, except for the records row, which is expressed in units. 1 + ADL (with difficulty in one or more activities of daily living).

**Table 2:** Results of the model evaluation with ST1, ST2, and ST3.

Strategy	Testing Proportion	Sensitivity Average	Sensitivity Error	Specificity Average	Specificity Error	Kappa Coefficient Average	Kappa Coefficient Error	Variable Interaction
ST1	0.1	0.6719	0.0038	0.5102	0.0037	0.0786	0.0025	No
	0.1	0.6537	0.0086	0.5271	0.0039	0.0800	0.0032	Yes
	0.2	0.6713	0.0048	0.5066	0.0027	0.0777	0.0014	No
	0.2	0.6594	0.0060	0.5222	0.0030	0.0803	0.0026	Yes
	0.3	0.6716	0.0036	0.5098	0.0026	0.0787	0.0010	No
	0.3	0.6611	0.0056	0.5213	0.0026	0.0802	0.0015	Yes
	0.4	0.6812	0.0043	0.5052	0.0024	0.0802	0.0010	No
	0.4	0.6728	0.0046	0.5153	0.0026	0.0823	0.0011	Yes
ST2	0.1	0.7317	0.0092	0.4717	0.0057	0.0918	0.0023	No
	0.1	0.7136	0.0061	0.477	0.0041	0.0855	0.0032	Yes
	0.2	0.7237	0.0062	0.4723	0.0049	0.0870	0.0028	No
	0.2	0.7215	0.0039	0.4935	0.0040	0.0990	0.0025	Yes
	0.3	0.7361	0.0036	0.4641	0.0030	0.0895	0.0022	No
	0.3	0.7206	0.0056	0.4787	0.0044	0.0903	0.0024	Yes
	0.4	0.7402	0.0052	0.4535	0.0041	0.0843	0.0008	No
	0.4	0.7274	0.0044	0.4752	0.0038	0.0907	0.0013	Yes
ST3	0.1	0.7589	0.0117	0.4358	0.0067	0.0916	0.0083	No
	0.1	0.7258	0.0073	0.455	0.0060	0.0864	0.0062	Yes
	0.2	0.7601	0.0069	0.4281	0.0039	0.0869	0.0026	No
	0.2	0.7401	0.0090	0.4489	0.0069	0.0869	0.0036	Yes
	0.3	0.768	0.0072	0.4124	0.0042	0.0814	0.0026	No
	0.3	0.7493	0.0061	0.4457	0.0032	0.0922	0.0031	Yes
	0.4	0.7729	0.0075	0.4210	0.0036	0.0877	0.0024	No
	0.4	0.7402	0.0076	0.4471	0.0071	0.0885	0.0028	Yes

## Data Availability

Data are available on request by writing to the corresponding authors.
